# Hospitalist Perceptions of Barriers to Lung Ultrasound Adoption in Diverse Hospital Environments

**DOI:** 10.3390/diagnostics11081451

**Published:** 2021-08-11

**Authors:** Anna M. Maw, P. Michael Ho, Megan A. Morris, Russell E. Glasgow, Amy G. Huebschmann, Juliana G. Barnard, Robert Metter, David M. Tierney, Benji K. Mathews, Edward P. Havranek, Mark Kissler, Michelle Fleshner, Barbara K. Burian, Elke Platz, Nilam J. Soni

**Affiliations:** 1 Division of Hospital Medicine, University of Colorado School of Medicine, Aurora, CO 80045, USA; robert.metter@cuanschutz.edu (R.M.); mark.kissler@cuanschutz.edu (M.K.); michelle.fleshner@cuanschutz.edu (M.F.); 2 Division of Cardiology, University of Colorado School of Medicine, Aurora, CO 80045, USA; michael.ho@cuanschutz.edu; 3 Adult and Child Consortium for Health Outcomes Research and Delivery Science (ACCORDS), University of Colorado School of Medicine, Aurora, CO 80045, USA; megan.a.morris@cuanschutz.edu (M.A.M.); russell.glasgow@cuanschutz.edu (R.E.G.); juliana.barnard@cuanschutz.edu (J.G.B.); 4 Division of General Internal Medicine, University of Colorado School of Medicine, Aurora, CO 80045, USA; amy.huebschmann@cuanschutz.edu; 5 Medical Education Department, Abbott Northwestern Hospital, Minneapolis, MN 55407, USA; David.Tierney@allina.com; 6 Department of Hospital Medicine, HealthPartners, Bloomington, MI 55420, USA; benji@umn.edu; 7 Department of Medicine, Denver Health, Denver, CO 80204, USA; ed.havranek@dhha.org; 8 Human Systems Integration Division NASA Ames Research Center, Mountain View, CA 94043, USA; barbara.k.burian@nasa.gov; 9 Cardiovascular Division, Brigham and Women’s Hospital, Harvard Medical School, Boston, MA 02115, USA; eplatz@bwh.harvard.edu; 10 Division of Pulmonary and Critical Care Medicine and Division of General & Hospital Medicine, University of Texas Health San Antonio, San Antonio, TX 78229, USA; sonin@uthscsa.edu

**Keywords:** lung ultrasound, implementation science, point-of-care ultrasound

## Abstract

Despite the many advantages of lung ultrasound (LUS) in the diagnosis and management of patients with dyspnea, its adoption among hospitalists has been slow. We performed semi-structured interviews of hospitals from four diverse health systems in the United States to understand determinants of adoption within a range of clinical settings. We used the diffusion of innovation theory to guide a framework analysis of the data. Of the 27 hospitalists invited, we performed 22 interviews from four hospitals of diverse types. Median years post-residency of interviewees was 10.5 [IQR:5-15]. Four main themes emerged: (1) There are important clinical advantages to LUS despite operator dependence, (2) LUS enhances patient and clinician experience, (3) Investment of clinician time to learn and perform LUS is a barrier to adoption but yields improved efficiency for the health system and (4) Mandated training and use may be necessary to achieve broad adoption as monetary incentives are less effective. Despite the perceived benefits of LUS for patients, clinicians and health systems, a significant barrier to broad LUS adoption is the experience of time scarcity by hospitalists. Future implementation strategies should focus on changes to the clinical environment that address clinician barriers to learning and adoption of new skills.

## 1. Introduction

In recent years, there has been increasing interest in integrating point-of-care ultrasound (POCUS), ultrasound that is performed and interpreted at the bedside by a treating clinician, into diagnostic pathways within internal medicine. In particular, point-of-care lung ultrasound (LUS) has emerged as an accurate and practical imaging modality for the assessment of undifferentiated dyspnea and for the monitoring of volume status in patients with heart failure [1,2,3,4,5,6,7,8]. LUS has been shown to have multiple advantages over the current first-line imaging modality, chest x-ray, including increased accuracy and avoidance of ionizing radiation in diagnosis of some of the most common causes of dyspnea [1]: pleural effusion [9], pneumonia [10], pulmonary edema [11] and pneumothorax [12]. The COVID pandemic has underscored the many clinical advantages of LUS, as it is an accurate diagnostic tool for the diagnosis and monitoring of COVID-pneumonia with the added potential benefit of reducing the risk of COVID exposure for radiology staff [13,14]. In light of the growing evidence of its utility, multiple professional societies now endorse LUS use in acute care settings [1,15].

Despite its many advantages and increasing availability, adoption of diagnostic POCUS applications, including LUS, remains low. Prior studies have found lack of access to equipment and training to be the biggest barriers to implementation [16]. However, given the falling costs of portable ultrasound machines and increasing opportunities for training, determinants of adoption are likely changing.

The purpose of this study was to understand how internal medicine hospitalists, internist physicians who care for hospitalized adults [17], perceived LUS as a clinical tool through the lens of diffusion of innovations (DOI) theory [18] in order to identify current determinants of implementation and develop strategies to facilitate use among hospitalists. In addition to understanding how environmental factors such as access to machines and training affect adoption, we also sought to identify determinants of use once those fundamental elements were in place. For this reason, we included settings in which clinicians had easy access to equipment and training. 

## 2. Methods

### 2.1. Study Design

#### Conceptual Frameworks

We used the DOI theory to frame our investigation [18]. DOI is a theory that seeks to explain how and at what pace new ideas and technology are adopted. DOI has been applied in numerous disciplines, particularly in the study of adoption of medical innovations by health systems [19]. This theory proposes that one of the elements that influences the spread of an innovation is the potential adopter’s perception of the innovation. Key attributes of innovations that can affect the rate of adoption and adopter categories per DOI are outlined in Table 1. Adopter categories classify individuals within a social system based on how and when they decide to adopt an innovation. Understanding adopter categories can aid in efforts to facilitate adoption. DOI also proposes that the social system or context influences the uptake of new technology. 

To capture elements of the environment that may affect adoption of LUS by hospitalists, we used the Pragmatic Robust Implementation and Sustainability Model (PRISM) to guide development of interview questions. PRISM is a pragmatic multi-level contextual model that includes relatively specific domains relevant to LUS and is tied to implementation outcomes in the RE-AIM framework [20,21,22]. The RE-AIM [22] framework was developed to promote external validity and equity in research on health interventions and assesses both implementation and effectiveness outcomes. The contextual domains of PRISM include known drivers of implementation [21] in the external environment (i.e., national policies, guidelines, and incentives) and the internal setting (i.e., multi-level organizational characteristics, perspectives, implementation and sustainability infrastructure). Use of PRISM has been recommended for the planning stages of implementation of health interventions to help identify determinants (i.e., barriers and facilitators) that will inform the creation and selection of implementation strategies, thereby enhancing adoption, implementation, and maintenance of evidence-based practices [20,22].

Use of theorical models and frameworks, like DOI and PRISM, is a recommended practice in implementation science [23,24] as they provide a lens with which to understand determinants of implementation and select strategies that are likely to facilitate implementation. In this study, we selected DOI to better capture the determinants of adoption at the clinician level and PRISM to capture elements of the environment that may influence adoption by clinicians.

### 2.2. Study Sample and Setting

This study was approved by the Institutional Review Board at University of Colorado. Verbal consent was obtained from all participants. We interviewed hospitalists from four diverse hospital settings in which LUS was used by some clinicians. Site 1 was a 700-bed private hospital in Minneapolis, Minnesota. Site 2 was a 465-bed private hospital in St. Paul, Minnesota. Site 3 was a 268-bed government hospital in San Antonio, Texas. Site 4 was a 700-bed safety net teaching hospital in San Antonio, Texas. Sites 2, 3 and 4 are University affiliated. All hospitals trained internal medicine resident physicians. Hospitalists from all sites were interviewed to capture their experiences, perceptions, and opinions regarding LUS use in their respective hospitals. We used purposeful sampling for initial study recruitment and snowball sampling to complete enrollment. Purposeful sampling is a non-random sampling technique that is used to recruit participants who can provide in-depth and detailed information about the phenomenon being studied. Snowball sampling occurs when enrolled study participants identify possible future study participants among individuals they know [25].

### 2.3. Data Collection

Between November 2020 and January 2021, the study team conducted semi-structured interviews with hospitalists at the four study sites to assess their perspectives on LUS implementation in their local setting. The interview questions (see Supplementary Materials) were guided by PRISM [20,21] in order to capture contextual factors that may affect the perception of LUS by hospitalists and evolved over the course of data collection. Participants were asked questions related to determinants of LUS for multiple indications including some questions specifically related to COVID as data collection occurred during the pandemic. Because the data suggested that there were many determinants of adoption unique to the pandemic and, in light of the decreasing impact of COVID in high resource inpatient settings relative to the time of data collection, responses to questions specific to COVID will be presented separately, so that the data and themes more representative of usual practice circumstances can be fully described and discussed here. Data acquired from questions addressing determinants of LUS adoption unique to the COVID pandemic will be presented in future writing. Qualitatively trained interviewers (AMM, MF and JGB) conducted all interviews by phone or video conferencing. Data collection continued until preliminary analyses indicated thematic saturation, when no additional themes were emerging from the interviews. 

### 2.4. Data Analysis

The interviews were audio recorded and transcribed verbatim. We applied DOI to develop a deductive coding framework [18,26]. We allowed for new codes to inductively arise during the data analysis. Members of the research team (AMM and RM) began the analysis by immersing in the data and then met to develop the initial coding framework and definitions based on the DOI domains. A subset of transcripts was independently coded, and the research team subsequently met to reconcile coding differences and further refine and develop the coding framework. This process continued until a final coding framework was agreed upon and finalized. A member of the research team (AMM) applied the framework to the remaining transcripts with a second member (RM) double coding 20% of the transcripts to ensure consistency in coding across the transcripts. All discrepancies were reconciled through consensus. The codebook and analysis were reviewed by another team member (MF) and a doctoral-trained qualitative expert (MAM). Coded data were analyzed within and across different hospitals to identify themes that represent the participants’ perceptions of the LUS adopter categories through the lens of DOI.

## 3. Results

Of the 27 hospitalists invited to interview, a total of 22 (15 adopters, 7 non-adopters) were enrolled and participated in interviews that lasted 30 to 45 min (Table 2). Median years post-residency of participants was 10.5 [IQR:5-15]. Recruited hospitalists had a broad spectrum of LUS experience ranging from novices to experts who routinely used LUS for diagnosis of multiple disease processes, including pneumothorax, pneumonia, pleural effusion, and pulmonary edema. Adopter status and training experience was determined by qualitative data. Participants were designated adopters if they considered diagnostic LUS a tool they had integrated into their usual care of patients. The site-specific data presented in Table 3 were collected in qualitative interviews of POCUS leaders from each site. Participants at site 1 completed a residency with an established three-year POCUS curriculum that required demonstration of LUS competency before residency graduation or completed a 7-day POCUS course in which participants received 7 full days of POCUS didactics and supervised scanning with LUS being one of the included applications. Some participants at sites 2 and 4 had completed or were in the process of completing the Society of Hospital Medicine (SHM) POCUS certificate of completion program which consists of on-line didactics, in person conferences with supervised hands-on scanning, completion of an imaging portfolio as well as a written and practical examination, with LUS being one of the required applications. The data fit well within the DOI framework and all attributes of innovation domains were used. 

### 3.1. Overview of Themes

Four main themes emerged from these data (Table 4): (1) There are important clinical advantages to LUS despite operator dependence, (2) LUS enhances patient and clinician experience, (3) Investment of clinician time to learn and perform LUS is a barrier to adoption but yields improved efficiency for the health system and (4) Mandated training and use may be necessary to achieve broad adoption as monetary incentives are less effective. Themes were similar across adopter status and study sites.

Participants perceived important clinical advantages of LUS including increased accuracy and expedited diagnoses. However, they acknowledged that ensuring LUS competency was a prerequisite to improving patient outcomes with its use. Some participants also perceived LUS as improving both patient and clinician experience.

Participants reported the investment of their personal time to become proficient and perform LUS exams was one of the biggest barriers to adoption but felt that adoption had important potential benefits for the health system (i.e., reduced length of stay and diagnostic testing). 

Despite both adopters and non-adopters acknowledging the benefits of LUS, only a minority of clinicians had adopted LUS, even in environments where portable or handheld ultrasound machines were easily accessible and training was incentivized. POCUS leaders interviewed felt that mandated training and use may be necessary to achieve broad implementation.

### 3.2. Important Clinical Advantages of LUS despite Operator Dependence

*Increased accuracy and expedited diagnosis:* Participants perceived multiple clinical advantages of LUS over chest x-ray including expedited diagnosis, improved accuracy, and reduced radiation exposure to patients. Many hospitalists felt LUS outperformed chest x-rays for multiple common diagnoses including decompensated heart failure, one of the most common reasons for hospitalization in adults. Hospitalist 1-3 said: *you can see pulmonary edema much better on ultrasound [than chest x-ray].*

Both users and nonusers of LUS reported expedited clinical decision-making was an important clinical advantage of LUS use. Hospitalist 2-3 said: *If I have a question ‘Why is this patient hypoxic?’ and I wanna know it now, I get my ultrasound, and I’m in their room, and I’d do it right there. Chest x-rays, even if I ordered it stat, will take maybe another 15, 20 min.*

*Perceived advantage of LUS among nonadopters:* All participants regardless of adoption status perceived potential benefits of LUS use. For example, hospitalist 1-5 who had undergone extensive LUS training for which he had received a monetary incentive, but had not yet integrated LUS into his practice said: *It’s a better tool than the stethoscope and if I were training today, I doubt I would be using only a stethoscope*. Another nonadopter, hospitalist 4-2 said: *I don’t think I need to be convinced. Honestly, I am convinced. It’s more how do I get the training?*

*Operator-dependence*: A concern expressed by some participants was that the accuracy of LUS is operator dependent and inadequate training could result in patient harm. Although this was acknowledged as a potential risk, it was not necessarily considered a reason to avoid adoption. Hospitalist 3-3 said: “*It could also be pretty inaccurate, just like the physical exam, just like a poorly done history. When it is more operator-dependent, more human-centered, it’s gonna have that variability. That’s never been a reason, in my mind, not to... That just means you need to train up to it and learn it and develop confidence*.”

### 3.3. Enhanced Patient and Clinician Experience:

Participants reported perception of improved patient experience and enhanced therapeutic rapport with LUS use, in part because patients received more time and attention from their clinician. Hospitalist 3-3 said: *It gives you more time with the patients, and they really feel like you’re examining them in a meaningful way.* Participants perceived LUS as also having a clinical advantage of offering an additional and powerful opportunity for patient education. Hospitalist 1-3 said: *I’m looking for a big pleural effusion that I’m gonna tap, I get to review that effusion with them [the patient], and they’re often like, "Whoa, my gosh." I say, "If you take your diuretics and you lay off salt, a lotta this can be avoided."*

Many clinicians reported LUS use improved their own practice experience. Hospitalist 3-3, who had some experience using LUS but was still in the process of deciding whether to adopt said: *[LUS] benefits you in the enjoyment of your job. [It] benefits the patients and their feeling of a therapeutic alliance… although it’s a technology, some of the … more human-centered aspects of it are, to me, the biggest advantages.* In addition, even when LUS findings didn’t change management, many participants reported it significantly reduced diagnostic uncertainty which they felt was valuable. Hospitalist 1-3 said: *It makes me have much more peace of mind when I’m making clinical decisions.* And gives them a feeling they are providing better care: Hospitalist 3-5 said: ”*I feel like it allows me to help care for my patients better.”*

Clinicians also reported enjoying the ability to answer urgent clinical questions quickly at the beside without relying on traditional imaging services which have an inherent delay. Hospitalist 3-3 said: *There’s a number of advantages that come with something being point-of-care that you control, as opposed to having to rely on others. If you have a need for a stat chest x-ray, that stat can be minutes or it could be an hour or sometimes longer. I think that level of control over the process, not being reliant on other people, being able to do it yourself, I think is huge*.

### 3.4. Investment of Clinician Time to Perform LUS Is a Barrier to Adoption but Yields Improved Efficiency for the Health System

*Time to learn and perform LUS*: Hospitalists describe the investment of clinician time as the most important disadvantage of LUS adoption. Even at sites 1 and 2 where training is easily available and even incentivized, the time clinicians needed to invest to attain proficiency, optimize efficiency and perform LUS once scanning efficiency was optimized was considered the most important barrier to adoption by both adopters and nonadopters. Hospitalist 2-1 said: *It comes down to time and you have to be extremely self-motivated. I’ve seen people whose enthusiasm flares up and then it washes away. It happens so, so commonly. That’s the reason why there’s only so few of us have been able to cross the finish line.*

Even after optimal efficiency is obtained, clinicians describe the extra time it takes at the bedside to perform a LUS exam is a barrier to LUS use. Hospitalist 3-1 said: *No. I think it’s really just the time I think is the biggest factor with it. Yeah, that’s really the biggest disadvantage I think.*

*Replacement of chest X-ray with LUS*: Multiple clinicians at sites 1 and 2 reported using LUS as initial imaging in patients with worsening respiratory status and reported greatly decreased use of chest x-rays since adopting LUS because they felt it was more accurate and helped them expedite appropriate management. Hospitalist 2-1 stated: *In the setting of a patient whose respiratory status is acutely worsening, I don’t even bother with chest x-ray. I usually just go with the ultrasound machine.*

Reducing the number of chest x-rays was perceived by many participants as one of the potential advantages of LUS implementation at a system level and consistent with high-value care. Hospitalist 1-1 said: *I do think there’s cost advantage if there’s a reduction in use … because x-ray is a multilevel thing, where somebody comes, takes the patient. Somebody takes the picture. Somebody reads the picture. Somebody uploads it. There’s like five people going in the background, giving a bunch of time … versus an ultrasound, you have the bedside person just going there, making a decision right there.*

*Improved efficiency for the health system:* Despite the additional cost of clinician time, many participants felt adoption of LUS could improve hospital efficiency by expediting and improving accuracy of diagnoses. Hospitalist 2-2 said: *Cost savings is clearly there as far as doing point-of-care ultrasound during my rounding and making a decision. It may shorten the length of stay, as far as being able to not wait a half day to get a CAT scan and get a CAT scan read if you are able to do the lung ultrasound and reassure yourself that the patient—that things are doing fine and they’re ready for discharge. It also, just the sheer cost of CAT scans is there, so I think there’s cost savings.*

### 3.5. Mandated Training and Use May Be Necessary to Achieve Broad Adoption as Monetary Incentives Are Less Effective

At site 1 and 2 where ultrasound equipment and training were easily accessible, participants reported only partial adoption. A large number of hospitalists in both groups had undergone training but still had not adopted LUS use in clinical practice. 

POCUS leaders at sites 1 and 2 reported using monetary incentives to encourage clinicians to train and perform POCUS. However, these incentives were perceived as having limited utility. Hospitalist 1-4: *It [monetary incentives] are not the solution. People that don’t want to do it, still don’t want to do it with money involved.*

Clinicians who had received training but had not yet adopted LUS stated they were more comfortable with other diagnostic tools and were not sure if their decision-making would be improved by LUS without a significant additional investment in time using LUS in clinical practice. Adopters speculated on the reasons why trained clinicians had not adopted LUS. Many adopters felt that curiosity, perseverance, and younger age predicted clinicians who were more likely to adopt LUS. POCUS leaders at sites 1 and 2 speculated that graduate medical education (GME) training requirements or a mandate from leadership would likely be necessary to increase uptake of LUS broadly to the early majority and late majority adopters.

Hospitalist 1-4 said: *You can’t actually get to the endpoint [integration of LUS into actual practice] until GME is at a longitudinal three-year point-of-care ultrasound integrated place.*

Hospitalist 2-6: I would anticipate institutionalizing, meaning this needs to be done.

## 4. Discussion

In this qualitative study of 22 hospitalists who practice in 4 diverse clinical settings, we have captured key perceptions of LUS through the lens of DOI that are likely to influence the rate of adoption by hospitalists. Overall, our findings suggest LUS was perceived by all study participants as offering relative advantages compared to standard tools including increased accuracy, expedited diagnosis, and improved patient and clinician experience. In addition, multiple participants from sites that had easy access to equipment and training reported that LUS had supplanted chest x-ray in their practice, a finding they perceived as consistent with high-value care. Although there have been studies demonstrating introduction of LUS can decrease the use of chest x-rays in intensive care units [27,28], this is a novel finding among hospitalists. 

Through the lens of DOI, many participant perceptions of LUS predict rapid adoption of LUS by hospitalists. However, our study shows that this is not the case. In terms of barriers encountered, our findings suggest that in environments in which equipment and training are not readily available, access to these necessary elements of adoption remain the most important barriers to adoption. This finding is consistent with previously published surveys on this topic [16]. In contrast, we found that even in environments in which the barriers of access to machines and training had been removed, only a minority of clinicians adopt. In these environments, the biggest barrier to adoption reported by participants is the clinician time required to attain proficiency and once proficiency is achieved, time to perform the exam. This is a novel finding that has important implications for future implementation efforts.

A possible explanation for these findings through the lens of DOI would be that LUS is highly “complex”; however, this is not consistent with what we know about this tool. Multiple studies have demonstrated that clinicians can achieve proficiency in performing LUS exams with very brief training [7,29] and LUS exams can be performed in less than 5 min by an experienced operator [4]. Indeed, even those who have adopted LUS say that they are unable to utilize it due to time constraints when patient volumes are high. This signals that it is perhaps the *environment* and not the intervention that poses the barrier to adoption. 

Our participants describe a practice setting in which a task that takes only a few extra minutes at the bedside, although perceived as inherently valuable on multiple levels and consistent with their professional values, is considered too time consuming to perform. This mandate to maximize efficiency at the expense of connecting with patients and incorporating new skills that are uniformly perceived as an improvement on traditional tools is striking. It suggests that the perceived scarcity of time within the current inpatient environment places physicians at odds with their professional values of performing well considered clinical decisions, life-long learning and building therapeutic rapport with patients. Further, this reality exists in an era of rapidly advancing medical knowledge and technology. Our findings underscore the current dilemma for individuals in clinical practice: how does a clinician evolve her practice to adhere to changing practice standards if the need to optimize productivity, often measured in relative value units, leaves no time to learn? 

Recent studies tying physician burnout to time scarcity [28] raise the question of whether decreased workloads that allow time for incorporation of new skills into practice may actually decrease the cost of care by both improving outcomes and health system efficiency. Reducing clinician burnout reduces its subsequent negative impact on a health system, including poor quality clinical decisions and physician turnover, which are costly [30,31]. The problem of perceived time scarcity also raises the question of how clinicians are to stay abreast of medical advancement if there is no cognitive space in their practice to do so and may in part explain the well documented lag in adoption of evidence-based practices [32].

We must also recognize that although time scarcity was the most commonly cited factor limiting uptake of LUS by hospitalists, it seems possible the word *time* may be a stand-in for a host of factors that serve as barriers to the acquisition of new knowledge and skills in clinical practice. Literature on human factors engineering and the cognitive sciences emphasizes ways that environmental design, cognitive load, attention demands, and task allocation are all elements that contribute to the clinician’s sense of being ‘pressed for time’ [33,34]. This expanded view of the barrier articulated by study participants has the advantage of promoting inquiry that will lead to a better understanding how these factors contribute to the adoption of health innovations by clinicians which in turn can be applied as targeted changes in infrastructure, culture, and procedures to address barriers to clinician learning and evidence-based practice.

Some participants felt mandating LUS use may be necessary to achieve uniform implementation across a department or institution, since monetary incentives were not perceived as effective. However, our findings underscore a current lack of alignment between clinician and health system incentives regarding LUS adoption. Given business models greatly influence decision-making in health care, health system leaders must be convinced of LUS’s value as measured by increased efficiency and reduced overall cost before investing in the infrastructure needed to facilitate robust LUS implementation. Until this is achieved, broad adoption by clinicians is likely to remain unrealized. 

### Limitations and Future Directions

There are important limitations to this study which must be recognized. First, our findings represent the viewpoints of hospitalists at 4 teaching hospitals in the United States. Hospitalists in rural, non-teaching hospitals or hospitals in other countries may have unique perspectives that were not captured by our study. Additionally, our 4 participating institutions had national POCUS leaders among their faculty which likely increased general awareness of LUS among local clinicians. Finally, although we interviewed several nonusers, early adopters were over-represented in our sample, and it should be emphasized that these early adopters represent a minority in their practice groups.

These data help us understand both hospitalist perspectives of LUS and environmental factors that influence their decisions to adopt LUS. These findings will allow us to consider implementation strategies that address the fundamental barriers in different environments. Additionally, we captured hospitalist attitudes and patterns of adoption that mirror the adopter categories proposed in DOI. This observation will allow us to create and test implementation strategies using audience segmentation messaging. However, the major finding of these data is hospitalists interviewed felt that time was such a limited resource in their practice that it precluded integration of LUS use despite acknowledging the multiple benefits to patient care. This suggests implementation strategies that target institutional buy-in and policy will be needed to achieve complete implementation.

## 5. Conclusions

The hospitalists interviewed perceive LUS as having important benefits for patients, clinicians and health systems. The time required to master and perform LUS was perceived to be an important barrier to its adoption by hospitalists. This finding highlights the crises of perceived time scarcity in clinical practice and its impact on the adoption of health innovations by clinicians. 

## Figures and Tables

**Table 1:** Diffusion of Innovations Theory Domains.

Attributes of Innovations	Description
**Relative Advantage**	Advantage offered over traditional approach or tools (strongest predictor of adoption)
**Compatibility**	Alignment of the intervention with values and needs of the group adopting it (positively correlated with rate of adoption)
**Complexity**	How difficult the innovation is to use (negatively correlated with rate of adoption)
**Trialability**	Degree to which an innovation may be used experimentally on a limited basis (positively correlated with rate of adoption)
**Observability**	Visibility of an innovation’s results to others (positively correlated with rate of adoption)
**Categories** **of Adopters**	**Description**
**Innovators**	Risk takers; role is to launch a new idea into the system
**Early Adopters**	Respected members of a group of potential adopters who are perceived as skilled in selecting new ideas that should be adopted
**Early Majority**	Need to see some peers successfully using an innovation prior to adopting it
**Late Majority**	Will adopt innovation if it becomes inconvenient to not adopt it
**Laggards**	Must be certain an innovation will not fail prior to adopting it

**Table 2:** Characteristics of Participants.

Study Participant and Site	Years Post-Residency	General ** POCUS Training in Which ^#^ LUS was an Included Application	Adopter Status
**Site 1**			
1-1	6	7-day course	Adopter
1-2	20	7-day course	Adopter
1-3	1	3-year residency curriculum	Adopter
1-4	15	** POCUS program founder	Adopter
1-5	6	7-day course	Non-adopter
**Site 2**			
2-1	10	Completed * SHM certifiation	Adopter
2-2	3	Residency and post-residency training	Adopter
2-3	5	Residency and post-residency training	Adopter
2-4	12	Completed * SHM certification	Adopter
2-5	4	Local certification process	Adopter
2-6	15	** POCUS program founder	Adopter
2-7	3	Residency, locally certified	Adopter
**Site 3**			
3-1	8	Residency and post-residency training	Adopter
3-2	25	2-day course	Non-adopter
3-3	18	2-day course	Non-adopter
3-4	17	** POCUS program founder	Adopter
3-5	12	Brief simulation training and bedside teaching from an expert	Non-adopter
**Site 4**			
4-1	2	Completing * SHM certification	Adopter
4-2	11	2-day course	Non-adopter
4-3	15	2-day course	Non-adopter
4-4	15	None	Non-adopter
4-5	9	Completing * SHM certification	Adopter

^#^ LUS, * SHM—Society of Hospital Medicine, ** POCUS—Point-of-Care Ultrasound.

**Table 3:** Description of Study Sites.

Site	Hospital Type	Number of Hospitalists	Number of Hospitalists Who Have Received Training	Number of Hospitalists Who Have Adopted LUS	Estimated Number of LUS Exams Performed Annually	Incentives Offered to Hospitalists	LUS Credentialing Process in Place	External Funding for Training
1	Private	78	78	30	3700	yes	no	yes
2	Private	100	70	10	1000	yes	yes	no
3	Government	24	19	3	312	no	no	no
4	Safety Net	68	16	3	468	no	no	no

**Table 4:** Themes and subthemes.

Theme	Subtheme	Exemplar Quotes
**Important clinical advantages of LUS despite operator dependence**	Increased accuracy and expedited diagnosis Perceived advantage of LUS among nonadopters Operator-dependence	***2-2:*** *I feel that I’m better able to pick up when I have dried out their lungs and gotten all the extra fluid out sooner with lung ultrasound than other modalities.* ***1-3:** If I come around on rounds at 9:00 a.m. and I say, "Let’s get a chest x-ray," the chest x-ray comes back at 11:00. Now I’m gonna give ’em one dose of diuretic if they’re overloaded. Versus if I come around at 9:00 a.m. and I check, and I see they’re overloaded. I can give ’em two doses. That’s humane, as opposed to making them be up all night peeing.* ***3-2:*** *I think ultrasound is much more convenient. You can do it yourself. The problem is comfort level with it. …I feel very comfortable with X-ray whereas ultrasound, it’s just not as familiar to me.* ***2-1:** I think it’s a sharp object. You can hurt yourself and your patients if you do it wrong.*
**Enhanced patient & provider experience**		***3-3:*** *By definition, you’re going to be at the bedside. You’re often, as you’re getting set up, gonna be making small talk about maybe not even the ultrasound but something else. They might be asking you questions about the ultrasound machine, and you’re answering them. It just creates a conversation, which can only, in my opinion, benefit everyone.* ***1-1:** just by doing an ultrasound, you have to be in that room for 10, 15 min. These days, if you watch any hospital environment, if you put up some kind of tracker device, the physicians barely spend eight minutes in a patient’s room. The rest of time goes into your coordination, your—the typing notes, all those things. I think that, first of all, you’re spending significantly more time in patient’s room, which they realize it, that this doctor’s been here for a while.*
**Investment of clinician time yields improved efficiency for the health system**	Time to master Time to perform Replacement of chest x-rays with LUS Improved efficiency for the health system	***4-1:****I think there’s interest, but again, it’s just a lot of—I think people get scared off by the commitment of having to go through all of that, the time that’s dedicated to it.* ***2-3:*** *We’re limited by time. We have many patients to see. Even if you want to ultrasound more, we’re limited by the amount of time we can spend at bedside, unfortunately.* ***1-1:****My x-ray use… over the last two years has dropped by 70 percent for acute shortness of breath in the hospital. Any time I get a call, and it’s like, "Oh, this patient is on oxygen, now four liters, and they’re short of breath," I immediately just go there with my ultrasound and try to figure things out rather than just— reflexively, previously I have always ordered an x-ray before I even leave my area where I’m working.* ***1-3:** Yeah, its [LUS] ability to replace x-ray and CT in many areas will be very helpful. Additionally, in an outpatient setting, I think that it has huge future implications for monitoring, preventing the need for people to be getting all these tests just to return to their primary doctor to discuss things. ….., I think, [LUS] has a real big future in reducing that need for multiple different imaging tests over the course of days.*
**Mandated training and use may be necessary to achieve broad adoption as monetary incentives are less effective**		***3-4:*** *I think the implementation has been slow for a host of reasons. What’s the incentive for anybody to take this on aside from being a good doctor or being able to not miss certain findings? There’s really not a lotta personal incentive…. How do you get to the guy who says, “I hate technology. I don’t wanna change. I’ve been doin’ my way for the last 20 years. What’s so much better about your way?” You could say your length of stay and your diagnoses will be better, but what does that really mean to me? The hospital already harasses me for so many other things. Length of stay is really their problem, not mine.* ***2-7:** The way that our group compensates is about 80 percent, roughly, base salary and then 20 percent that’s production-based in some way or another. A component of that production is RVU driven. I think a point-of-care lung gets 2.4 RVU … It’s not a huge incentive, I would say. I think there are probably easier ways to add some to your bottom line.*

## Data Availability

The data presented in this study are available on request from the corresponding author.

## Supplementary Materials

The following are available online at https://www.mdpi.com/article/10.3390/diagnostics11081451/s1, Supplementary Material File S1. Interview guide for Hospitalists.
